# Supplementary figures and images for: The organization of leukotriene biosynthesis on the nuclear envelope revealed by single molecule localization microscopy and computational analyses
Source: PLoS One. 2019 Feb 8;14(2):e0211943. doi: 10.1371/journal.pone.0211943 (PMC6368329; doi:10.1371/journal.pone.0211943)

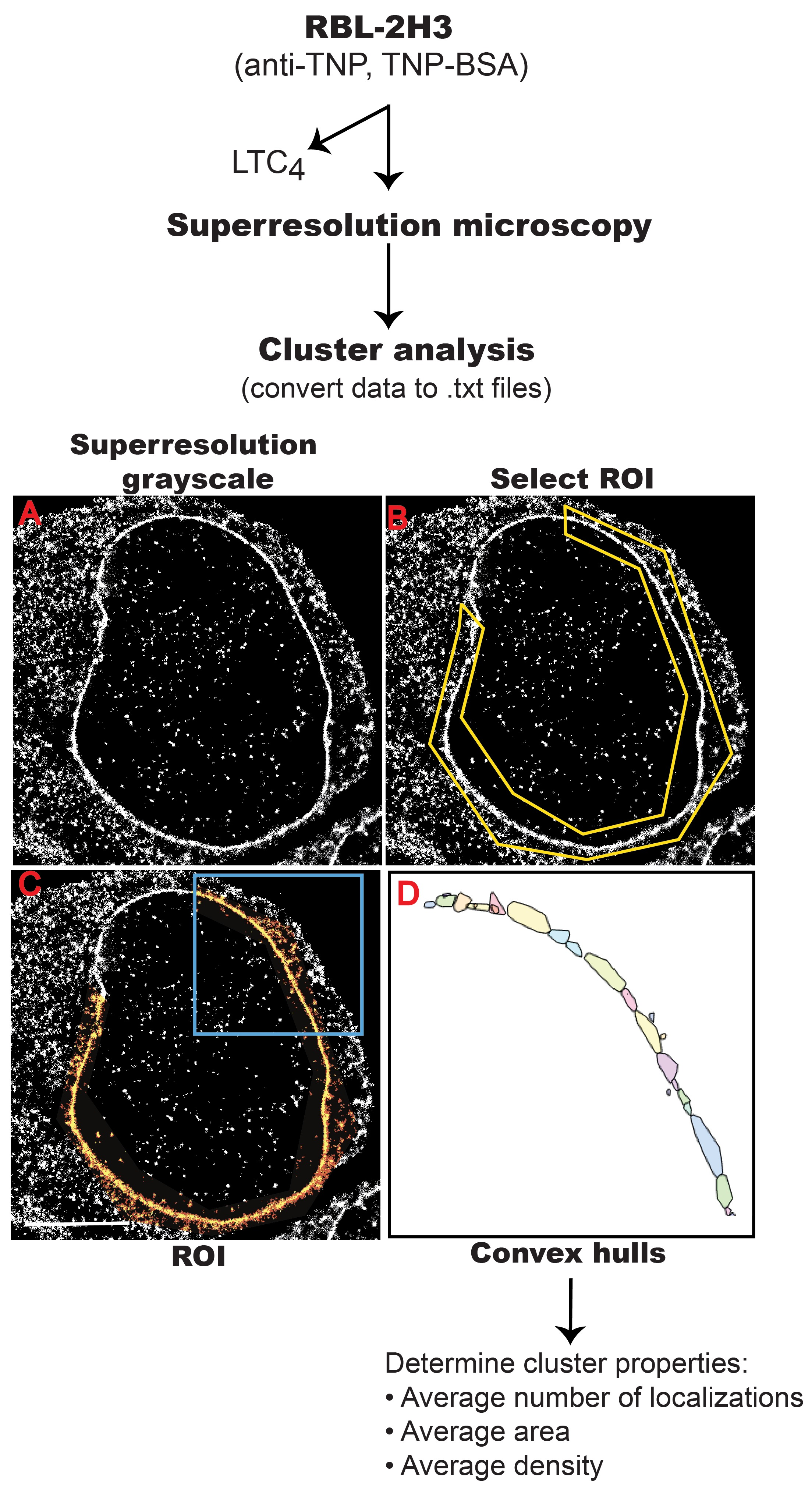

Supplement: S1 Fig — RBL-2H3 mast cells were primed with anti-TNP antibody followed by crosslinking of FcεR1 by addition of TNP-BSA. Media was removed from each well and analyzed for LTC4. Cells were fixed, permeabilized, and prepared for molecular imaging. STORM localization lists were converted to tab-delimited text files. Image panels illustrate the process. (A) STORM image of FLAP localizations in an activated mast cell. (B) A region of interest (ROI) is drawn around the nuclear envelope FLAP localizations. (C) Localizations inside the ROI (orange) are clustered by unbiased cluster analysis. (D) The convex hull of points in a cluster (inset area from C) defines the cluster area, from which properties are calculated including number of localizations, area and density. For 5-LO, the entire nucleus and perinuclear region were included in the ROIs because 5-LO is cytoplasmic in unstimulated cells and thus the nuclear envelope is not apparent. Scale bar = 4 μm. (TIF) [file pone.0211943.s001.tif]

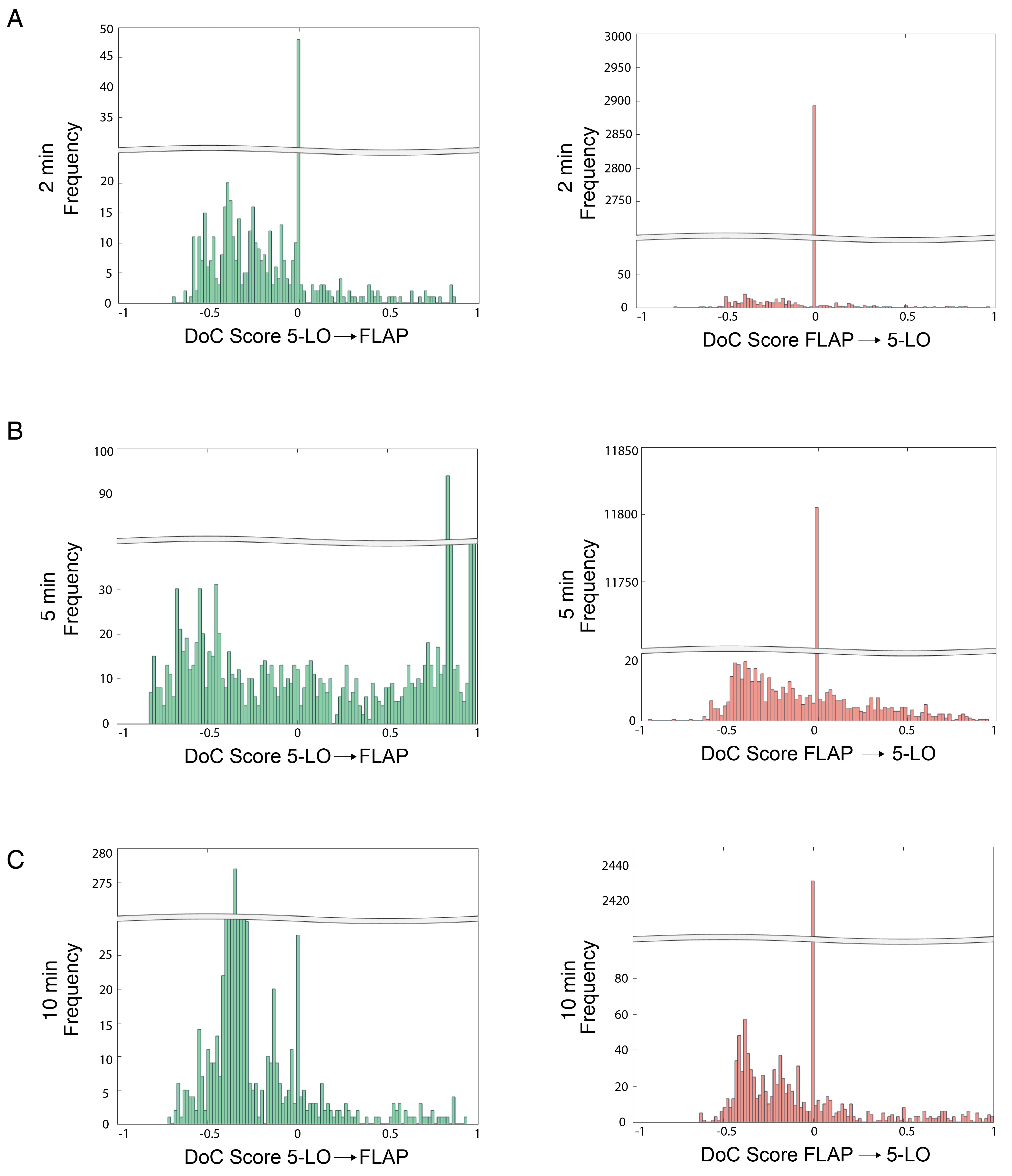

Supplement: S2 Fig — Localization data was collected by two-color dSTORM and analyzed with ClusDoC. The cells shown in Fig 2 were used to calculate DoC scores. (A) Histograms of DoC scores of all molecules for 5-LO (green) and FLAP (red) at 2min, (B) 7min, (C) 10 min. (TIF) [file pone.0211943.s002.tif]

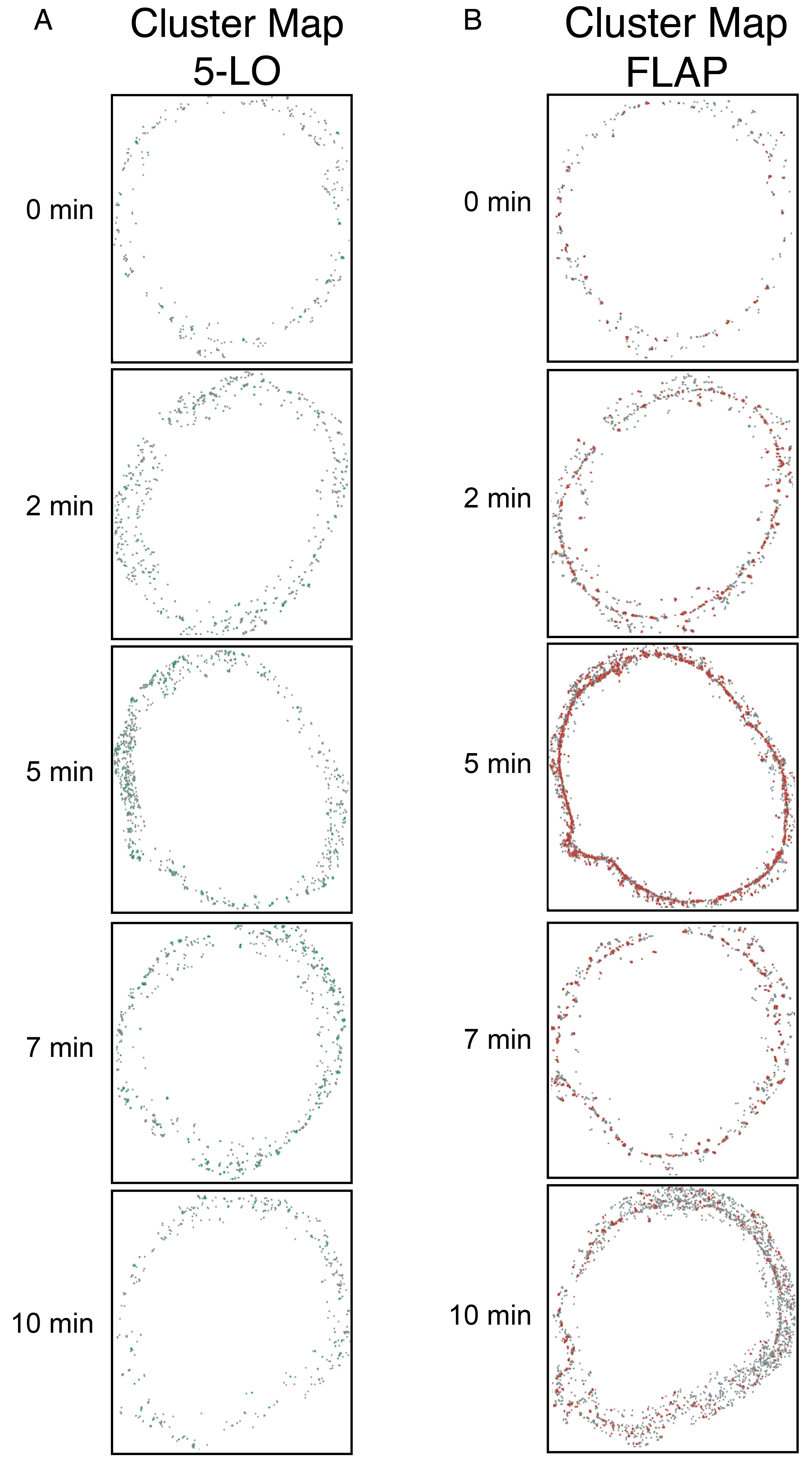

Supplement: S3 Fig — RBL-2H3 cells were primed with anti-TNP IgE then activated with TNP-BSA for 0, 2, 5 and 10 min. Localization data was collected by two-color dSTORM and analyzed with ClusDoC. Cluster maps for 5-LO (A, green) and FLAP (B, red) from representative cells from Fig 2 over time were generated. Nonclustered localizations are colored gray. (TIF) [file pone.0211943.s003.tif]

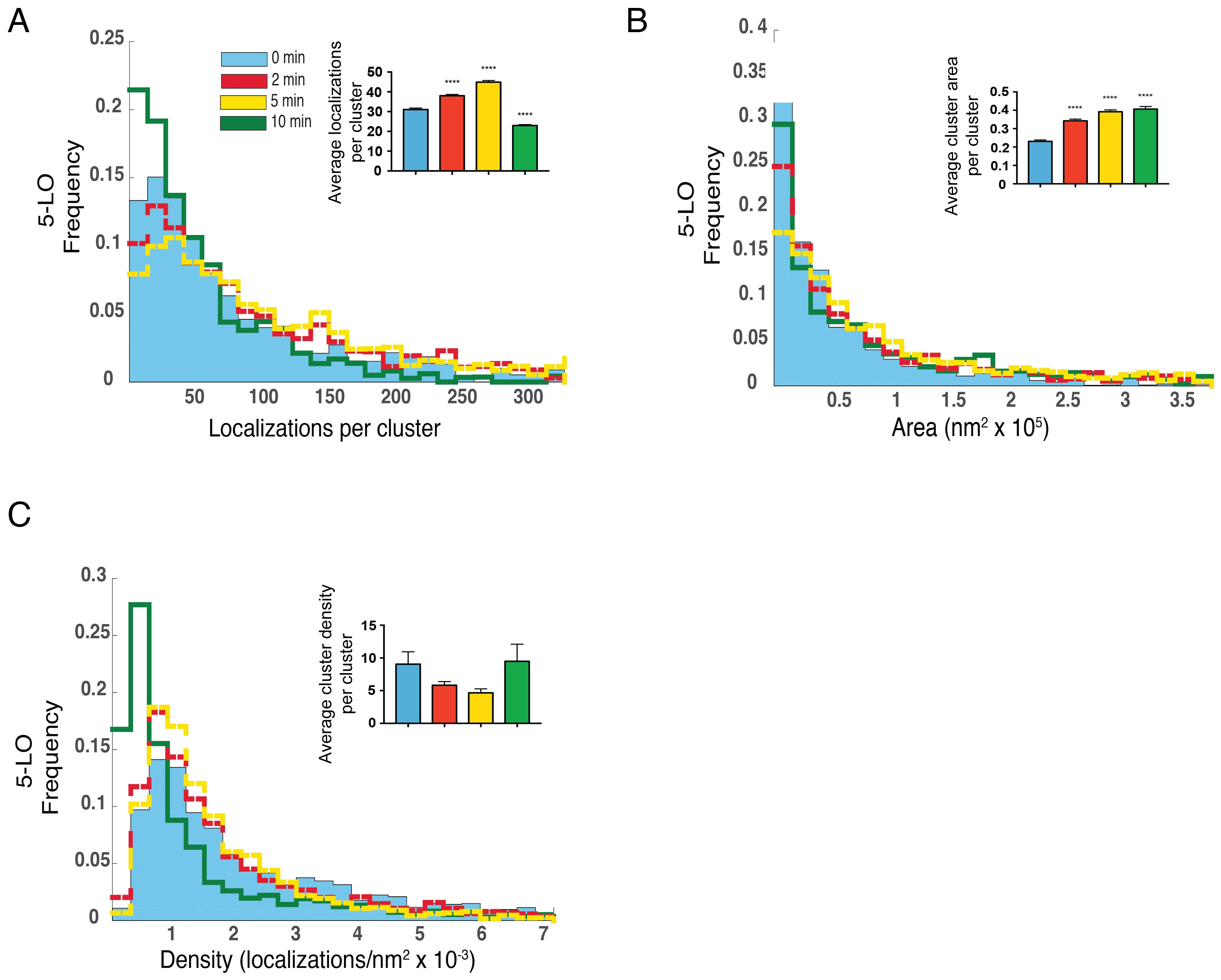

Supplement: S4 Fig — RBL-2H3 cells were primed with anti-TNP IgE then activated with TNP-BSA for 0, 2, 5 and 10 min and analyzed as shown S1 Fig. Cells were imaged with conventional STORM and cluster properties were analyzed with unbiased cluster analysis. (A-C) Normalized point-weighted histograms with inset bars showing mean ± SEM for (A) number of localizations, (B) cluster areas and (C) cluster densities. One-way ANOVA with Bonferroni post hoc test was performed to determine significance, indicated by ****p < 0.0005. At least 3 separate experiments collected between 10 and 30 cells. (TIF) [file pone.0211943.s004.tif]

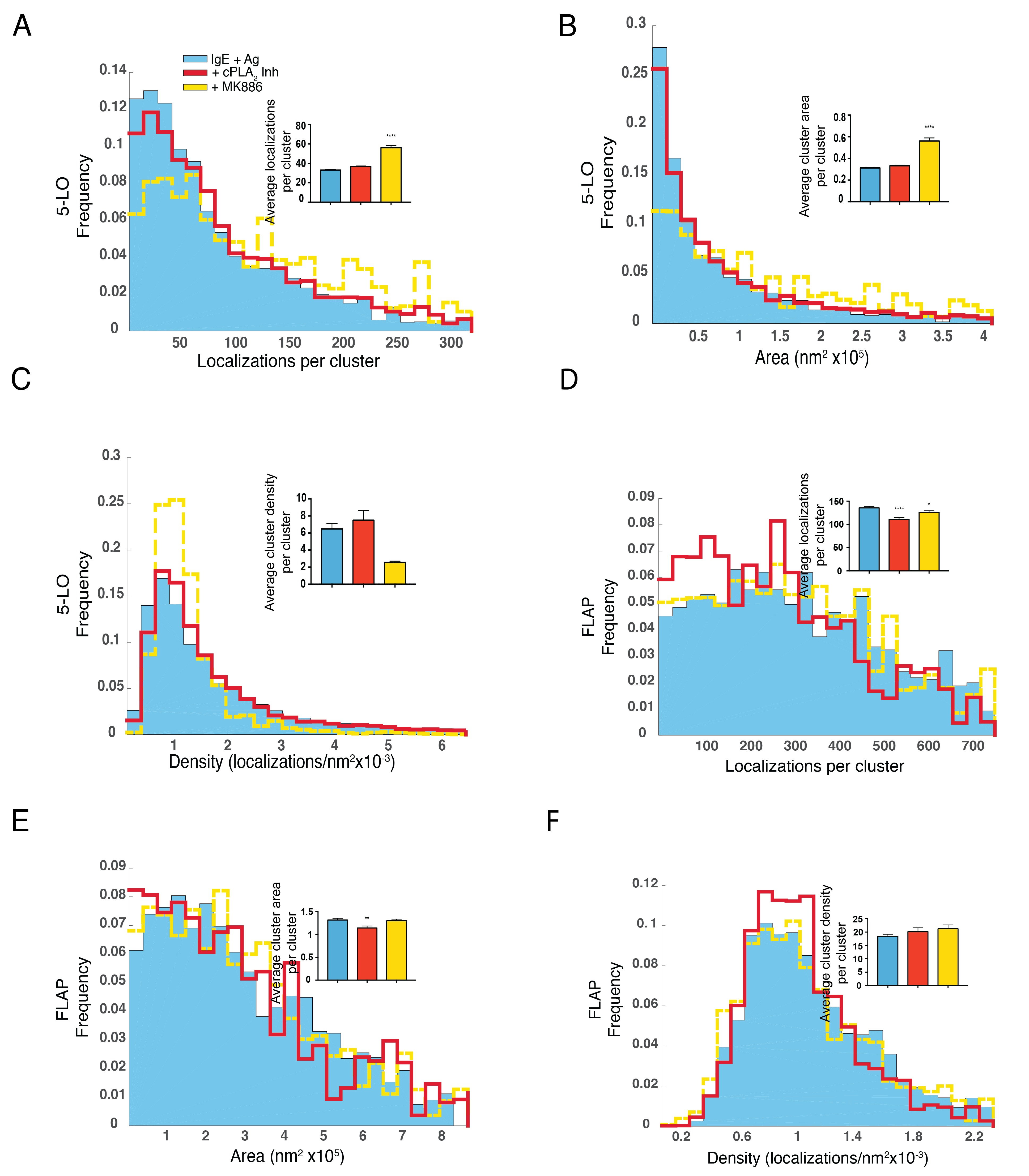

Supplement: S5 Fig — RBL-2H3 cells were incubated with or without cPLA2 Inh or MK886, and then primed with anti-TNP IgE. They were then stimulated by the addition of TNP-BSA for 7 min. The cells were imaged with conventional STORM, and cluster properties were analyzed with unbiased cluster analysis. (A-F) Normalized point-weighted histograms with inset bars showing mean ± SEM for (A,D) number of localizations, (B,E) cluster areas and (C,F) cluster densities for 5-LO and FLAP, respectively. The area shaded blue represents localizations in cells primed and activated for 7 min. The solid red line represents cells incubated with cPLA2 Inh and primed and activated. The dotted yellow line represents cells incubated with MK886 and primed and activated. One-way ANOVA with Bonferroni post hoc test was performed to determine significance, indicated by *p < 0.05 and ***p = 0.0005. At least 3 separate experiments collected between 10 and 30 cells. (TIF) [file pone.0211943.s005.tif]
